# *n*-3 Fatty Acid Supplementation and Leukocyte Telomere Length in Patients with Chronic Kidney Disease

**DOI:** 10.3390/nu8030175

**Published:** 2016-03-19

**Authors:** Anne Barden, Nathan O’Callaghan, Valerie Burke, Emile Mas, Lawrence J. Beilin, Michael Fenech, Ashley B. Irish, Gerald F. Watts, Ian B. Puddey, Rae-Chi Huang, Trevor A. Mori

**Affiliations:** 1 School of Medicine and Pharmacology, Royal Perth Hospital, University of Western Australia, Perth 6000, Western Australia, Australia; valerie.burke@uwa.edu.au (V.B.); emilie.mas@sa.gov.au (E.M.); lawrie.beilin@uwa.edu.au (L.J.B.); gerald.watts@uwa.edu.au (G.F.W.); ian.puddey@uwa.edu.au (I.B.P.); trevor.mori@uwa.edu.au (T.A.M.); 2 CSIRO Food and Nutrition, Adelaide 5000, South Australia, Australia; nathan.OCallaghan@csiro.au (N.O.C.); Michael.Fenech@csiro.au (M.F.); 3 Department of Nephrology and Transplantation, Fiona Stanley Hospital, Murdoch 6150, Western Australia, Australia; Ashley.Irish@health.wa.gov.au; 4 Telethon Kids Institute, Subiaco 6008, Western Australia, Australia; Rae-Chi.Huang@telethonkids.org.au

**Keywords:** neutrophils, telomere length, oxidative stress, *n*-3 fatty acids, coenzyme Q10

## Abstract

DNA telomere shortening associates with the age-related increase cardiovascular disease (CVD) risk. Reducing oxidative stress, could modify telomere erosion during cell replication, and CVD risk in patients with chronic kidney disease (CKD). The effect of *n*-3 fatty acids and coenzyme Q10 (CoQ) on telomere length was studied in a double-blind placebo-controlled trial in CKD. Eighty-five CKD patients were randomized to: *n*-3 fatty acids (4 g); CoQ (200 mg); both supplements; or control (4 g olive oil), daily for 8 weeks. Telomere length was measured in neutrophils and peripheral blood mononuclear cells (PBMC) at baseline and 8 weeks, with and without correction for cell counts. Main and interactive effects of *n*-3 fatty acids and CoQ on telomere length were assessed adjusting for baseline values. F_2_-isoprostanes were measured as markers of oxidative stress. There was no effect of *n*-3 fatty acids or CoQ on neutrophil or PBMC telomere length. However, telomere length corrected for neutrophil count was increased after *n*-3 fatty acids (*p* = 0.015). Post-intervention plasma F_2_-isoprostanes were negative predictors of post-intervention telomere length corrected for neutrophil count (*p* = 0.025).The effect of *n*-3 fatty acids to increased telomere length corrected for neutrophil count may relate to reduced oxidative stress and increased clearance of neutrophils with shorter telomeres from the circulation. This may be a novel mechanism of modifying CVD risk in CKD patients.

## 1. Introduction

Telomeres are chromatin structures that cap the ends of chromosomes and preserve genome stability. At a cellular level, loss of telomeric DNA eventually leads to cell death or senescence. A major factor contributing to telomere damage is oxidative stress that promotes telomere erosion during cellular replication and stimulates synthesis of inflammatory cytokines [1,2]. Telomere shortening has been associated with the age-related increase in risk of chronic conditions such as cancer and cardiovascular disease (CVD). Patients with chronic kidney disease (CKD) and those receiving dialysis have a greatly increased risk of CVD. There is data from a longitudinal study of patients with stable coronary heart disease suggesting shorter baseline telomere length is associated with lower glomerular filtration rate (GFR) [3]. Patients receiving haemodialysis have shorter telomeres and accelerated telomere shortening compared with the general population [4,5]. Telomere attrition associates with inflammation, low fetuin–A levels and increased mortality in CKD patients undergoing haemodialysis [6].

Telomere length is in part determined by cellular telomerase activity. Telomerase is a ribonucleoprotein that synthesises telomeres and can compensate for the loss of telomeres during cell division. Telomerase activity in hematopoietic progenitor cells determines the telomere length of circulating leukocytes. Mature neutrophils and monocytes differ from lymphocytes in that they do not undergo further cell division as they circulate and they are devoid of telomerase activity.

Population studies have shown that there is substantial variability in the rate of telomere shortening that is independent of chronological aging, suggesting that telomere loss may be a modifiable factor [7,8]. A number of lifestyle and dietary factors have been examined as possible determinants of telomere shortening in cross-sectional trials as well as animal studies. Smoking and obesity associate with telomere shortening while dietary restriction, increasing dietary fibre or anti-oxidants, or reducing fat or protein intake protect against telomere shortening [9]. Other factors that may affect telomere length include leukocyte cell counts that positively associate with telomere length [10,11,12]. This may be particularly relevant in patients with CKD whose neutrophil cell counts have been shown to be elevated, likely as a result of increased apoptosis [13]. Interventions aimed at reducing oxidative stress have been suggested as a possible way of inhibiting telomere shortening.

Studies in humans have shown that oxidative stress, as assessed by plasma and urinary F_2_-isoprostanes, is reduced by supplementation with *n*-3 fatty acids [14,15,16]. Another potential modifier of oxidative stress is coenzyme Q10 (CoQ) an intracellular antioxidant that protects membrane phospholipids, mitochondrial membrane protein, low-density lipoprotein and lymphocyte DNA from free radical-induced oxidative damage [17,18,19]. As oxidative stress is regarded as a major contributor to risk of CVD in patients with CKD [20], the implementation of interventions using *n*-3 fatty acids and/or CoQ are therefore considered possible modifiers of oxidative stress that might inhibit telomere shortening and have the potential to modify CVD risk. The only other study examining the effect of *n*-3 fatty acids on oxidative stress in predialysis patients was uncontrolled, of short duration (2 weeks), with small numbers of patients (*n* = 5) and assessed oxidative stress using a method that is known to be non-specific. [21]

In a randomized controlled trial that examined the main and additive effects of *n*-3 fatty acids and coenzyme Q10 (CoQ) on cardiovascular risk in patients with CKD we showed that *n*-3 fatty acid supplementation reduced blood pressure, heart rate and plasma triglycerides [22]. This report examines the effects of *n*-3 fatty acids and CoQ on telomere length of DNA from neutrophils and mononuclear cells of patients CKD from that study.

## 2. Volunteers and Methods

### 2.1. Participants

Eighty five men and women aged 25–75 years with chronic renal impairment (defined as an estimated (e) GFR >15 and <60 mL/ min/1.73 m^2^ and serum creatinine <350 mmol/L), were recruited between March 2004 and October 2006 from the renal units of Royal Perth, Sir Charles Gairdner and Fremantle Hospitals, in Perth, Western Australia,. Exclusion criteria included: diabetes; angina pectoris; major surgery, a cardiovascular event or symptoms of CVD <3 months; BP >170/100 mmHg; liver disease; nephrotic syndrome (proteinuria >3 g/day or protein/creatinine ratio >300 mg/mmol); and haemoglobin <110 g/L. Patients were also excluded if they were smokers; regularly took non-steroidal anti-inflammatory or immunosuppressive drugs; nitrates; the phosphodiesterase inhibitor sildenafil; ate >1 fish meal/week or regularly took fish oil supplements; or if they consumed an average >4 standard alcoholic drinks/day. Antihypertensive and lipid-lowering medication were not exclusion criteria. All patients gave informed written consent and the trial was approved by the ethics committee of Royal Perth Hospital, Sir Charles Gairdner and Fremantle Hospitals. The trial was registered with the Australian and New Zealand Clinical Trials Registry (ACTRN012605000088640) and conducted in accordance with the Declaration of Helsinki.

### 2.2. Study Design

The trial was double-blind and placebo-controlled. Patients maintained their usual dietary habits during a 3-week familiarization period. After baseline measurements were obtained they were stratified by age and BMI, and randomly allocated by the statistician using computer-generated random numbers to one of four study groups: *n*-3 fatty acids (4 g daily, Omacor, Solvay Pharmaceuticals, Pymble, NSW, Australia), CoQ (200 mg daily, Blackmores Australia (Balgowlah, NSW, Australia), the two combined (*n*-3 fatty acids + CoQ), or control (4 g daily olive oil, Cardinal Health Australia, Braeside, Victoria, Australia), for 8 weeks, The allocation sequence was concealed using numbered containers until interventions were assigned at the time of enrolment by study personnel not involved in the process. Capsules of *n*-3 fatty acids contained 460 mg eicosapentaenoic acid (EPA, 20:5 *n*-3), 38 mg docosapentaenoic acid (DPA, 22:5 *n*-3) and 380 mg docosahexaenoic acid (DHA, 22:6, *n*-3). Patients took 2 × 1 g *n*-3 fatty acid or control, and 2 × 50 mg CoQ or placebo capsules, twice daily with meals. They were asked to maintain their usual diets and lifestyle during the intervention. Compliance with the supplements was monitored by measurement of platelet fatty acids and plasma CoQ.

### 2.3. Measurement of Leukocyte Telomere Length and Plasma F_2_-Isoprostanes

Blood samples and a 24 h urine collection were obtained at baseline and at the end of the eight week intervention. Peripheral blood mononuclear cells (PBMC) and neutrophils were isolated from venous blood by density gradient centrifugation using Ficoll-Paque (Pharmacia Biotech, Uppsala, Sweden), cell counts were measured using the Technicon H1 Analyser (Bayer Diagnostics, Sydney, Australia) and neutrophils and PBMC were prepared for cryopreservation in fetal bovine serum (FBS) plus 10% DMSO, prior to storage in liquid nitrogen until required. DNA isolation used a QIAGEN DNeasy Kit with minor modifications to prevent DNA oxidation [23]. Absolute telomere length (aTl) of DNA from PBMC or neutrophils was measured by determining the number of TTAGGG hexamer repeats using quantitative real-time PCR (qPCR) as previously described [22]. The method is based on the qPCR technique described by Cawthon [24] to measure relative telomere length (rTL) but modified by using a standard curve generated with serial dilution of a synthetic telomere repeat sequence oligomer. The standard curve was used to convert rTL values to aTL data which are reported as kb/diploid genome. The assays were done in duplicate and the inter- and intra-experimental variation was less than 7%. Plasma F_2_-isoprostanes were measured by gas chromatography-mass spectrometry as previously described [25]. High sensitivity C-reactive protein (hs-CRP) was measured using a high sensitivity monoclonal antibody assay (Dade Behring Marburg GmbH, Marburg, Germany) as previously described [26].

### 2.4. Statistical Analysis

Analyses included only participants who completed the trial. Sample size was based on the primary endpoint of blood pressure [22]. The study had an 80% power to detect a 3 mm Hg difference in ambulatory systolic blood pressure at *p* < 0.05. Post-intervention data were analysed using SPSS15.0 (SPSS Inc., Chicago, Illinois, IL, USA) or SAS 9.0 (SAS Inc., Chicago, Illinois, IL, USA) with general linear models adjusting for baseline values and assessing main and interactive effects of *n*-3 fatty acids and CoQ. Baseline measures were compared by one-way analysis of variance. Significance levels were adjusted for multiple comparisons by the Tukey test. Values are means (SEM) or geometric mean (95% confidence interval (CI)). In order to account for any potential confounding effects of the treatments on leukocyte cell counts that may have influenced DNA telomere length [10,11,12], analyses were performed on neutrophil DNA telomere length corrected for neutrophil cell count or PBMC DNA telomere length corrected for PBMC cell count. Regression models were used to explore the relationship between plasma F_2_-isoprostanes and telomere length.

## 3. Results

Eighty-five patients aged 56.5 ± 1.4 years, with BMI 27.3 ± 0.5 kg/m^2^, supine BP 125.0 ± 1.7/72.3 ± 0.9 and eGFR 35.8 ± 1.2 mL/min/1.73 m^2^ were randomized to the four groups. Seventy-four patients completed the study. The consort diagram for this clinical trial has been published [22]. DNA from neutrophils and PBMCs was available from 73 of the 74 patients: control (*n* = 15); *n*-3 fatty acids (*n* = 19); CoQ (*n* = 21) and *n*-3 fatty acids + CoQ (*n* = 18). Bodyweight and eGFR were similar at baseline in the 4 groups and did not change significantly during the intervention [22]. Antihypertensive medication was taken by 72 of the 74 patients and did not change throughout the study. At baseline, 74% of patients allocated to *n*-3 fatty acids were taking angiotensin converting enzyme inhibitors compared with 75% of those allocated to the control oil [22].

The study showed good compliance with *n*-3 fatty acid and CoQ intake. Platelet long chain *n*-3 fatty acids (20:5 + 22:5 + 22:6) increased from baseline by 6.4% ± 1% (*p* < 0.001) in the *n*-3 fatty acid group and by 5.4% ± 1.1% (*p* < 0.001) in the *n*-3 fatty acid + CoQ group compared with the control and CoQ groups. Plasma CoQ levels increased in the CoQ group by 3260 ± 351 nmol/L (*p* < 0.001) and by 2114 ± 286 nmol/L (*p* < 0.001) in the *n*-3 fatty acid+CoQ group, compared with the control and *n*-3 fatty acid groups. There were no reported side effects of the supplements in any group.

At baseline neutrophil counts in the groups were: Controls, 3.8 ± 0.2 × 10^9^ cells/L; *n*-3 fatty acid, 3.1 ± 0.2 × 10^9^ cells/L; CoQ, 3.9 ± 0.3 × 10^9^ cells/L; and *n*-3 fatty acid + CoQ. 4.0 ± 0.2 × 10^9^ cells/L. Post intervention neutrophil counts adjusted for baseline were not different between the groups (*p* = 0.174). At baseline, PBMC counts were not different between the groups: Controls, 2.0 ± 0.1 × 10^9^ cells/L; *n*-3 fatty acid, 2.2 ± 0.1 × 10^9^ cells/L; CoQ, 2.2 ± 0.1 × 10^9^ cells/L; and *n*-3 fatty acid + CoQ. 2.2 ± 0.1 × 10^9^ cells/L and were unaffected by the interventions.

### 3.1. Telomere Length at Baselinein Patients with CKD

At baseline, neutrophil telomere length was not different between the groups (Table 1). However, PBMC telomere length was significantly shorter in the *n*-3 fatty acid + CoQ group compared with the group allocated to take CoQ alone (*p* = 0.03, Table 1). In the groups combined, baseline neutrophil telomere length was not significantly associated with PBMC telomere length (*r* = 0.173, *p* = 0.149). There were no significant associations between neutrophil or PBMC telomere length and age, BMI, eGFR, gender, platelet *n*-3 fatty acids, hs-CRP or plasma F_2_-isoprostanes at baseline.

### 3.2. Effect of n-3 Fatty Acids and CoQ on Telomere Length

After 8 weeks of intervention there were no significant main or interactive effects of *n*-3 fatty acids or CoQ on neutrophil or PBMC telomere length (Table 1). Post-intervention telomere length in neutrophils and PBMC were not significantly related.

### 3.3. Effect of n-3 Fatty Acids and CoQ on Telomere Length Corrected for Cell Count

**Figure 1:**
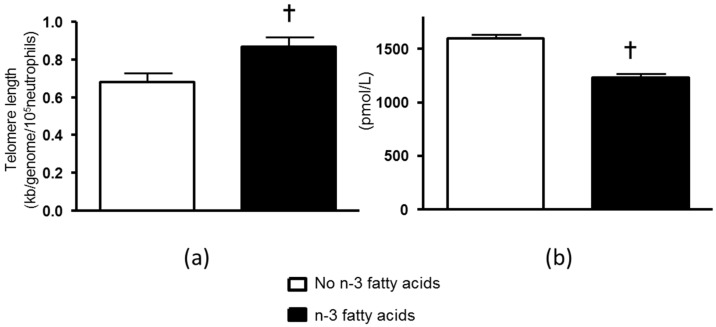
(**a**) Post intervention neutrophil telomere length corrected for neutrophil count and adjusted for baseline values in patients taking *n*-3 fatty acids compared with those not taking *n*-3 fatty acids. † *p* = 0.015 for main effect of *n*-3 fatty acids post-intervention adjusted for baseline values; (**b**) Post-intervention plasma F_2_-isoprostanes in patients taking *n*-3 fatty acids compared with those not taking *n*-3 fatty acids. † *p* < 0.001 for main effect of *n*-3 fatty acids post-intervention adjusted for baseline values. Values are means ± SEM.

Neutrophil telomere length was not significantly associated with neutrophil count at baseline but was positively associated with neutrophil count after the intervention (*r* = 0.317, *p* = 0.006). In order to account for any potential confounding effects of the treatments on leukocyte cell counts that may have influenced DNA telomere length [10,11,12], analyses were performed on neutrophil DNA telomere length corrected for cell count. Neutrophil telomere length corrected for neutrophil count was not significantly different between the groups at baseline, but was increased in a main effect analysis that compared patients after *n*-3 fatty acids (0.19 ± 0.17 kb/genome/10^5^ cells, *p* = 0.015) with those that did not receive *n*-3 fatty acids, adjusting for baseline values (Table 1, Figure 1A). The effect of *n*-3 fatty acids remained significant after adjusting for age, gender and BMI (*p* = 0.008). In contrast, CoQ did not significantly alter neutrophil telomere length after correcting for neutrophil cell count (−0.10 ± 0.07, *p* = 0.17, for main effect). There was no significant interaction between *n*-3 fatty acids and CoQ that affected neutrophil telomere length (Table 1).

There were no significant correlations between PBMC telomere length and PBMC count at baseline or after the intervention. At baseline, PBMC telomere length corrected for PBMC count was significantly lower in the *n*-3 fatty acid + CoQ group compared with CoQ alone (*p* = 0.03, Table 1). Post-intervention, there was no effect of *n*-3 fatty acids or CoQ on PBMC telomere length corrected for PBMC count (*p* = 0.73) and (*p* = 0.90), respectively, after adjusting for baseline values. There was no significant interaction between *n*-3 fatty acids and CoQ that affected PBMC telomere length (Table 1).

### 3.4. Relationship between Plasma F_2_-Isoprostanes and Telomere Length

At baseline plasma F_2_-isoprostanes were not different between the groups: Controls, 1602 (CI 1423, 1780) pmol/L; *n*-3 fatty acids, 1714 (CI 1539, 1888) pmol/L; CoQ, 1448 (CI 1346, 1550) pmol/L; and *n*-3 fatty acids + CoQ. 1479 (CI 1325, 1632) pmol/L. Post-intervention plasma F_2_-isoprostanes were: Controls, 1658 (CI 1553, 1764) pmol/L; *n*-3 fatty acids, 1215 (CI 1119, 1312) pmol/L; CoQ, 1533 (CI 1443, 1624) pmol/L; and *n*-3 fatty acids + CoQ. 1253 (CI 1156, 1350) pmol/L. In main effects analysis there was a significant reduction in F_2_-isoprostanes after *n*-3 fatty acids (Figure 1B), but no effect of CoQ on plasma F_2_-isoprostanes. In regression analysis, after adjusting for baseline values post-intervention neutrophil telomere length corrected for neutrophil count was negatively related to post-intervention plasma F_2_-isoprostanes (β = −0.242, *p* = 0.025). The model explained 21.2% of the variation in neutrophil telomere length corrected for neutrophil count. There was no significant relationship between post-intervention neutrophil telomere length corrected for neutrophil count and post-intervention eGFR, blood pressure or hs-CRP. There were no significant associations between post-intervention PBMC telomere length corrected for PBMC count and post-intervention plasma F_2_-isoprostanes or post-intervention eGFR, blood pressure or hs-CRP.

## 4. Discussion

This study examined the main and interactive effects of *n*-3 fatty acids and CoQ on neutrophil and PBMC telomere length in patients with CKD. There was no effect of *n*-3 fatty acids or CoQ on telomere length of neutrophils or PBMC. However, *n*-3 fatty acid but not CoQ supplementation, was associated with a significant increase in neutrophil telomere length after correcting for neutrophil count. The effect remained significant after adjusting for age, gender and BMI. The increase in telomere length (corrected for neutrophil count) was negatively associated with post-intervention plasma F_2_-isoprostanes. Telomere length of PBMC corrected for PBMC count was not affected by *n*-3 fatty acids or CoQ.

Previous report in several different populations have shown a positive association between leukocyte count and telomere length [10,11,12]. In our population with CKD there was a significant relationship between neutrophil telomere length and neutrophil count post intervention. However, there was no association between baseline PBMC or neutrophil count with telomere length. Baseline telomere length measured in neutrophils was poorly correlated with telomere length of PBMC suggesting that there may be different factors affecting telomere length of these cell types. The majority of cells in the PBMC fraction are lymphocytes (60%–70%) with monocytes contributing 10%–15% of cells. Unlike lymphocytes, mature neutrophils and monocytes do not undergo further cell division and are devoid of telomerase activity. Therefore, changes in telomere length in these cells likely reflects that of their hematopoietic progenitor cells [27]. Interestingly, it has been reported that *n*-3 fatty acids given during pregnancy increased the percentage of cord blood CD34^+^ progenitor cells [28]. These myeloid progenitor cells are critical for the development of neutrophils. Whether *n*-3 fatty acids in our intervention affected neutrophil progenitor cell phenotype in the same manner and whether this is, in some way, associated with telomere length is not known.

Our results show that telomere length corrected for neutrophil count was increased after *n*-3 fatty acids. Correction of telomere length for cell count can be justified on the grounds of previous reports indicating telomere length is positively correlated with cell count [10,11,12]. As mature neutrophils do not have telomerase, the increase in telomere length (corrected for neutrophil count) may be due to increased neutrophil clearance (efferocytosis) possibly due to senescence that renders neutrophils unable to respond to chemoattractants or degranulate. Senescence is accompanied by an increase in the chemokine receptor CXCR4 on the neutrophil surface that homes neutrophils back to the bone marrow where they undergo apoptosis [29]. This could result in a pool of circulating neutrophils that have less telomere erosion that is highlighted when we correct for neutrophil counts. Critical to this process of neutrophil clearance are specialised proresolving mediators of inflammation (SPM) that derive from *n*-3 fatty acids. These substances promote resolution of inflammation and a return to homeostasis by increasing neutrophil efferocytosis. In this respect resolvin D1 (RvD1), an SPM derived from DHA, has been shown to increase expression of CXCR4 and reduce lung injury after a challenge with lipopolysaccharide [30]. We have shown that *n*-3 fatty acids but not CoQ increased plasma RvD1 in this trial [31]. This could suggest that when the immune system is directed from an inflammatory state to resolution and a return to homeostasis, telomere length is affected. *n*-3 Fatty acids could also affect telomere length by reducing oxidation in the telomere TTAGGG sequence. In support of this concept, we have shown that *n*-3 fatty acids but not CoQ significantly reduced plasma F_2_-isoprostanes (markers of lipid oxidation) in this study [32] and report herein a significant negative relationship between plasma F_2_-isoprostanes and telomere length (corrected for neutrophil count) accounting for a significant proportion (21.2%) of the variation in telomere length.

The effect of *n*-3 fatty acids on telomere length corrected for neutrophil count remained significant after adjustment for BMI, gender and age, and was not related to changes in blood pressure, GFR or hs-CRP. Our study was of a relatively short duration and renal function was not altered by either intervention. We have previously reported reductions in blood pressure, heart rate and serum triglycerides after *n*-3 fatty acid supplementation in this study [22]. The sample size for this study was based on the primary endpoint of blood pressure rather than neutrophil and PBMC telomere length that were measured as secondary outcomes. Therefore, a larger trial of longer duration of *n*-3 fatty acid supplementation will be required to determine whether effects on neutrophil telomere length associate with a reduction in CVD in patients with CKD.

Our study is the first to measure and compare telomere length in neutrophils and mononuclear cells after *n*-3 fatty acid supplementation in patients with CKD. Other studies examining the effect of *n*-3 fatty acids on telomere length include a prospective cohort study in aging patients with coronary heart disease that showed an inverse association between *n*-3 fatty acids measured in blood at baseline and the rate of telomere shortening over 5 years [33]. There have been two reports of controlled studies of *n*-3 fatty acids on telomere length. A small randomised controlled study of *n*-3 fatty acids *versus* linoleic acid in elderly individuals with mild cognitive impairment did not show any effect of *n*-3 fatty acids (~2 g/day) given for 6 months on telomere length, but reported that increased erythrocyte DHA associated with reduced telomere shortening [34]. A larger randomised controlled trial in healthy men and women compared the effects of two doses of *n*-3 fatty acids (1.25 g/day and 2.5 g/day) for 4 months on lymphocyte telomere length, telomerase activity, and oxidative stress assessed from measurement of plasma F_2_-isoprostanes [35]. The trial demonstrated a significant fall in plasma F_2_-isoprsoatnes after *n*-3 fatty acids with no effect on lymphocyte telomere length or telomerase activity. However, telomere length was inversely associated with *n*-6:*n*-3 fatty acid ratio [35]. The reduction in oxidative stress is consistent with the data from our study that showed a significant reduction in plasma F_2_-isoprostanes after *n*-3 fatty acids [32]. In addition, our study provides evidence that post-intervention plasma F_2_-isoprostanes are negatively related to telomere length accounting for a significant proportion (21.2%) of the variation. These findings suggest that reducing oxidative stress may prevent telomere shortening as has been suggested in the literature [36,37,38,39].

Post-intervention PBMC telomere length was not significantly influenced by *n*-3 fatty acids or CoQ either before or after adjustment for PBMC count. The differences observed between DNA obtained from neutrophils and PBMC could in part reflect the relative turnover of the cell types. Neutrophil half-life is estimated to be 6–8 h [29] whereas the half-life of monocytes and lymphocytes range is 1–3 days [40] and up to 80 days [41], respectively. Our findings suggest that any effect of *n*-3 fatty acids on telomeres may differ according to the cell type and turnover. In a recent study of samples collected from human cadavers, telomere length of leucocytes was compared with that of 11 different tissues. The study showed that leukocyte telomere length was poorly correlated with telomere length in a variety of tissues [42]. We measured telomere length in a mixed population of PBMC that included monocytes as well as T and B lymphocytes. Therefore, any changes in PBMC telomere length would likely have been influenced by changes in the proportion of monocytes and lymphocyte subsets, that have been shown to be very heterogeneous in terms of telomere length and telomerase activities [43] Our ability to detect changes in telomere length of PBMC may also have been influenced by the large proportion of patients who were treated with ACE inhibitors. These agents are known to suppress lymphocyte function [44], and to reduce markers of DNA oxidation [45] in patients with CKD.

We found no effect of CoQ on neutrophil or PBMC telomere length or oxidative stress assessed by plasma F_2_-isoprostanes in contrast to a previous study in a rabbit model of atherosclerosis that showed CoQ protected against oxidation of lymphocyte DNA [46]. However, that study provided a relatively high dose of CoQ (25 mg/kg) to animals that were fed a high fat diet. A small open trial in humans showed that CoQ given as 6 mg/kg/day for 4 weeks reduced lymphocyte DNA 8-hydroxydeoxy-guanosine [47].

## 5. Conclusions

In conclusion, *n*-3 fatty acid supplementation in patients with CKD was associated with an increase in neutrophil telomere length that was corrected for neutrophil count. The finding that post-intervention telomere length negatively associated with plasma F_2_-isoprostanes, further suggests the effect of *n*-3 fatty acids on telomere length may be due, in part, to a reduction in oxidative stress. Long term studies of the effects of *n*-3 fatty acids in CKD patients are required to confirm the present findings and determine if changes in telomere length may be associated with a reduction in CVD risk in patients with CKD.

## Figures and Tables

**Table 1:** Neutrophil and peripheral blood mononuclear cells (PBMC) telomere length before and after correction for cell counts at baseline and after 8 weeks intervention.

	Control (*n* = 15)	*n*-3 FA (*n* = 19)	CoQ (*n* = 21)	*n*-3 FA + CoQ (*n* = 18)	ANOVA at Baseline (*p* Value)		Interaction (*p* Value)
					Main Effects (*p* Value)		
					*n*-3 FA	CoQ	
Neutrophil telomere length (kb/genome)							
Baseline	163.4 ± 21.5	159.1 ± 16.1	181.2 ± 14.3	149.7 ± 12.3	NS		
Post	166.9 ± 11.5	160.9 ± 10.3	155.6 ± 9.8	184.8 ± 10.6	29.3 ± 14.6	24.6 ± 14.7	
					(*p* = 0.28)	(*p* = 0.54)	*p* = 0.1
Neutrophil telomere length corrected for neutrophil count (kb/genome/10^5^ cells)							
Baseline	0.61 ± 0.10	0.82 ± 0.12	0.97 ± 0.18	0.55 ± 0.06	*p* = 0.25		
Post	0.72 ± 0.08	0.91 ± 0.70	0.63 ± 0.07	0.82 ± 0.08	0.19 ± 0.07	−0.10 ± 0.07	
					(*p* = 0.015)	(*p* = 0.17)	*p* = 0.97
PBMC telomere length (kb/genome)							
Baseline	103.9 ± 11.6	86.5 ± 11.4	114.1 ± 9.8	75.0 ± 8.2 *	*p* = 0.03		
Post	107.1 ± 11.6	101.9 ± 10.2	96.5 ± 9.7	107.3 ± 10.5	10.5 ± 14.7	5.1 ± 14.5	
					(*p* = 0.48)	(*p* = 0.73)	*p* = 0.46
PBMC telomere length corrected for PBMC count (kb/genome/10^5^ cells)							
Baseline	1.06 ± 0.14	0.89 ± 0.13	1.18 ± 0.14	0.66 ± 0.11 *	*p* = 0.03		
Post	1.16 ± 0.15	0.95 ± 0.14	0.99 ± 0.13	1.08 ± 0.14	−0.64 ± 0.14	−0.02 ± 0.14	
					(*p* = 0.9)	(*p* = 0.73)	*p* = 0.29

Values expressed as mean ± SEM. *n*-3 FA = *n*-3 fatty acid supplementation. Post = post-intervention data adjusted for baseline values. Baseline measures were compared by one-way ANOVA. General linear models were used to assess main effects and interactions of *n*-3 fatty acids and Coenzyme Q10 (CoQ) on post-intervention values adjusted for baseline value. Significance levels were adjusted for multiple comparisons by the Tukey test. * *p* < 0.05 for baseline difference between patients allocated to *n*-3 fatty acids + CoQ compared with those allocated to CoQ alone.
